# The association of serum vitamin D level and neonatal respiratory distress syndrome

**DOI:** 10.1186/s13052-023-01415-w

**Published:** 2023-01-30

**Authors:** Weili Liu, Pingping Xu

**Affiliations:** Department of Neonatology, Cangzhou Central Hospital, 16 Xinhua Road, Cangzhou, 061000 Hebei China

**Keywords:** Neonatal respiratory distress syndrome (NRDS), Vitamin D, 25(OH)D_3_, Multivariate logistic regression analysis, Risk factors

## Abstract

**Background:**

Neonatal respiratory distress syndrome (NRDS) is a critical disease in premature infants. Vitamin D plays an important role in promoting the development of fetal lung structure and the formation of pulmonary surfactants. This study aimed to investigate the correlation between the serum 25(OH)D_3_ level in the cord blood of premature infants and the prognosis of NRDS.

**Methods:**

This retrospective study recruited 82 preterm infants (gestational age 28-36 weeks) diagnosed with NRDS as the NRDS group, and 82 non-NRDS preterm infants as the control group, respectively. The diagnostic efficiency of 25(OH)D_3_ on NRDS was revealed by receiver operating characteristics curve (ROC) analysis. Enzyme linked immunosorbent assay (ELISA) was performed to evaluate the 25(OH)D_3_ level in the serum of the cord blood in preterm neonates. The NRDS risk indicators were identified by the multivariate logistic regression analysis.

**Results:**

Cord blood 25(OH)D_3_ levels were significantly lower in NRDS preterm infants than control group infants. 25(OH)D_3_ levels in cord blood can be used to predict NRDS in preterm infants. In addition, 25(OH)D_3_ levels in cord blood were positively correlated with Apgar score (1 min/5 min) and negatively correlated with oxygen support/CPAP duration in preterm infants with NRDS. 25(OH)D_3_ in cord blood <57.69 nmol/L (24 ng/ml), gestational age <31 weeks, birth weight <1.86 kg, Apgar score (1 min) <7 and Apgar score (5 min) < 8 were independent risk factors for NRDS.

**Conclusion:**

25(OH)D_3_ level is an independent risk factor for NRDS in preterm infants.

**Supplementary Information:**

The online version contains supplementary material available at 10.1186/s13052-023-01415-w.

## Introduction

Neonatal respiratory distress syndrome (NRDS) is a common respiratory disease among preterm infants [1]. As a serious complication, NRDS often causes symptoms such as shortness of breath, cyanosis, respiratory failure, and dyspnea several hours after birth, which seriously threatens the life of newborns [2]. Although the endotracheal pulmonary surfactant therapy has improved patient outcomes [3], NRDS remains a serious disease with high mortality among preterm infants. In survivors, NRDS may be further complicated by bronchopulmonary dysplasia, a chronic lung disease characterized by impaired alveolar development and inflammation [4]. Using pulmonary surfactant to treat NRDS is expensive and may require repeated dosing, as exogenous surfactant does not increase production of local surfactant [5]. Therefore, exploring the risk factors of NRDS in premature infants is of great value for the prevention, diagnosis and treatment of NRDS.

Vitamin D is a class of fat-soluble steroid derivatives with a wide range of biological activities. After vitamin D enters the blood circulation, it undergoes two hydroxylation actions in the liver and kidneys to form 25(OH)D_3_ and 1,25-dihydroxy vitamin D_3_ (1,25-(OH)_2_D_3_), respectively [6]. 25(OH)D_3_ has an increased half-life and relatively stable nature and is recognized as a key factor in measuring vitamin D reserves in the body [7]. Vitamin D is thought to play a crucial role in embryonic development, cell growth and differentiation, including lung development and fetal lung maturation, while vitamin D deficiency may aggravate neonatal lung disease in preterm infants [8]. It has been reported that vitamin D inhibits the profibrotic effects of transforming growth factor-β1 in lung fibroblasts and epithelial cells in rats [9]. In addition, a physiologically relevant BALB/c mouse model of vitamin D deficiency showed deficits of lung functions [8]. Adham et al. reported that low 25(OH)D_3_ level was far frequent among Egyptian preterm neonates and vitamin D deficiency was an independent risk factor for respiratory distress syndrome in preterm neonates [10]. However, there is not much research on the correlation between NRDS and vitamin D.

This study aimed to explore the relationship between neonatal serum 25(OH)D_3_ level and NRDS, which might provide evidence for clinical diagnosis and management of NRDS.

## Methods

### Participants

The retrospective study was performed to investigate the relationship between respiratory distress syndrome of preterm infants and umbilical cord serum vitamin D level. Eighty-two children with NRDS were selected for the study group and 82 preterm infants without NRDS were selected as the control group. This research was approved by the institutional review board of Cangzhou Central Hospital. Informed consent was obtained from all parents of the infants. Inclusion criteria and exclusion criteria are shown in Table 1. Infants received parenteral nutrition upon admission to the neonatal intensive care unit (NICU). The general intravenous nutrition of premature infants with NRDS includes: amino acids, fat emulsion, glucose, water-soluble and fat-soluble vitamins, electrolytes, and trace elements. Vitamin D was included in this intravenous nutrition. The data of venous nutrition duration of the infants were shown in Table S1.

**Table 1:** Inclusion and exclusion criteria in this study

Inclusion criteria	Exclusion criteria
*•* Singleton *•* Birth gestational age 28-36 weeks *•* Admission to NICU within 24 hours after birth *•* Cord blood samples collected at birth *•* Informed consent obtained from parents *•* Clinical diagnosis of NRDS Medical records completed^a^	*• *Congenital and chromosomal anomalies *• *Severe liver and kidney dysfunction *• *Amniotic fluid aspiration syndrome *• *Presence of adenomatous malformation of lung, diaphragmatic hernia and genetic defects related to surfactant *• *Infants moved away during the study period *• *Mother’s therapy that could affect vitamin D level

^a^According to the Montreux standard for neonatal acute respiratory distress syndrome (2017 Edition)

### Oxygenation index (OI) evaluation

Oxygenation index (OI) was calculated at the first mechanical ventilation within 24 hours after infants' admission to the NICU. $OI=\frac{\left(Mean Airvay Pressure\right)\times \left(Arterial Oxygen Partial Pressure\right)}{(Inhaled Oxygen Concentration) \times 100}$. Preterm infants in the NRDS group were divided into three groups according to their OI values: severe group (OI ≥ 16, *n*=18), moderate group (oxygen index 8 ≤ OI < 16, *n*=39) and mild group (4 ≤ OI< 8, *n*=25).

### Hypoxia assessment

Arterial blood gas analysis and the Apgar scale were used to assess hypoxia in preterm infants. Neonates admitted to the NICU were first assessed with the Apgar scale. The Apgar scale mainly includes five parts: skin color, heart rate, respiration, muscle tone and movement, and reflex, with 2 points for each part. The higher the score, the better the condition of the newborn. Arterial blood gas analysis was performed on the neonates after initial assessment with the Apgar scale. Arterial partial pressure of oxygen (PaO2), partial pressure of carbon dioxide (PaCO2) and pH were used to further assess neonatal asphyxia. PaO2<50 mmHg, PaCO2>60 mmHg, pH<7.20, BE<-5.0 mmol/L were indicators of neonatal hypoxemia, hypercapnia and metabolic acidosis.

### Enzyme-linked immunosorbent assay (ELISA)

Vitamin D levels (25‐hydroxyvitamin D [25(OH)D] concentration) were evaluated by ELISA. Commercial kits (DIAsource ImmunoAssays, Louvain‐la‐Neuve, Belgium) were used for the collection of the cord blood and further analysis of 25(OH)D_3_ levels in the neonates.

### Statistical analysis

The statistical analysis was performed using SPSS version 20. Data were presented as mean ± standard deviation and percentages. Categorical variables were compared using the Student t test, χ2, and F‐test (analysis of variance). Correlation between serum 25(OH)D_3_ concentration and number of parameters were analyzed by the Pearson correlation coefficient. *p* < 0.05 was considered statistically significant.

## Results

### Baseline characteristics of the study subjects

Eighty-two preterm infants diagnosed with NRDS were enrolled as the NRDS group, and 82 non-NRDS preterm infants were selected as negative control group. Demographic characteristics of the participants in this study were shown in Table 2. We found that there was no significant difference in gender, gestational age and weight at birth between two groups (*p*>0.05). However, Apgar score (1 min) and Apgar score (5 min) were remarkably lower in NRDS group (*p*<0.05) compared to the control group. The incidence of asphyxia in NRDS group has also increased (*p*<0.05). There was no statistical difference between mothers’ age, parity, delivery method (vaginal delivery or caesarian section), gestational diabetes mellitus, and gestational hypertension between the two groups (*p*>0.05). The NICU treatment between the two groups were compared in Table S1. The statistical differences between the two groups in continuous positive airway pressure ventilation (CPAP) duration, duration of oxygen support, length of hospital stay, and venous nutrition duration were identified (*p*<0.05).

**Table 2:** Demographic and clinical characteristics of the preterm babies with (NRDS) and without (Control) neonatal respiratory distress syndrome

Characteristics	Study group		*p* value
	Control (*n*=82)	NRDS (*n*=82)	
Gender			
Male	49 (59.8%)	44 (53.7%)	0.529
Female	33 (40.2%)	38 (46.3%)	
Gestational age (weeks)	32.7±2.8	31.9±3.2	0.283
Birth weight (Kg)	2.15±0.51	1.97±0.63	0.106
Apgar score (1 min)	8.14±1.39	6.76±1.45	< 0.001
Apgar score (5 min)	8.96±0.75	7.26±1.46	< 0.001
Asphyxia			
Yes	3 (3.7%)	21 (25.6%)	< 0.001
No	79 (96.3%)	61 (74.4%)	
NRDS classification			
Mild	-	25 (30.5%)	-
Moderate		39 (47.6%)	
Severe		18 (21.9%)	
Maternal age (years)	28.4±4.2	29.7±4.6	0.417
Parity, %			
0	42 (51.2%)	34 (41.5%)	0.236
1	36 (43.9%)	39 (47.6%)	
≥ 2	4 (4.9%)	9 (10.9%)	
Delivery			
Vaginal delivery	42 (51.2%)	31 (37.8%)	0.156
Caesarian section	40 (48.8%)	51 (62.2%)	
Gestational diabetes mellitus			
Yes	30 (36.6%)	37 (45.1%)	0.266
No	52 (63.4%)	45 (54.9%)	
Gestational hypertension			
Yes	9 (10.9%)	16 (19.5%)	0.128
No	73 (89.1%)	66 (80.5%)	

Values were expressed as n (percentage, %) or mean ± SD. *p* values for each group were derived from Mann–Whitney test. Chi-square test or Fisher’s exact test was used for assessing distribution of observations or phenomena between different groups

### *Cord blood serum 25(OH)D*_*3*_* level between the NRDS and control groups*

**Fig. 1:**
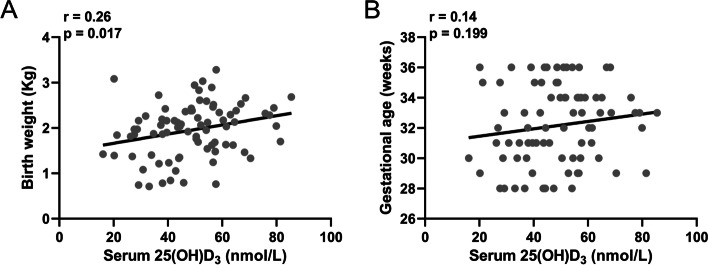
Spearman correlation analysis of 25(OH)D_3_ in cord blood with birth weight (**A)** and gestational age (**B)** in preterm babies with neonatal respiratory distress syndrome

**Fig. 2:**
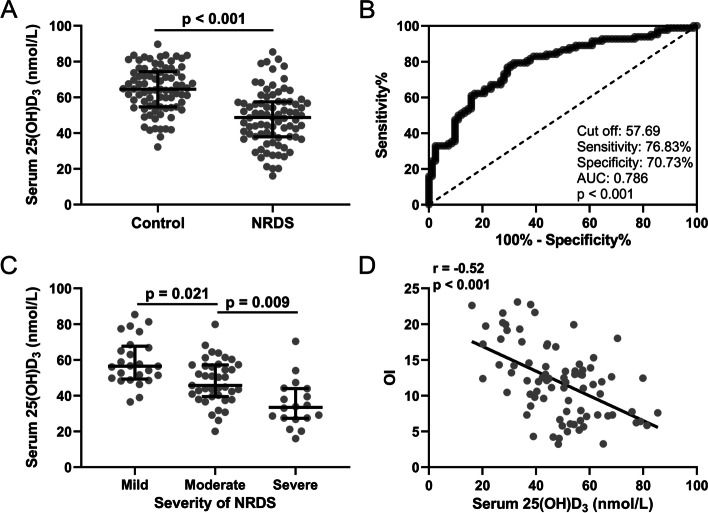
**A** Comparisons of serum levels of 25(OH)D_3_ of the NRDS group and Control group. Data were shown with median with quarterback rang. Mann Whitney test. **B** ROC analysis of serum 25(OH)D_3_ in cord blood for the prediction of NRDS in preterm infants. **C** Comparisons of 25(OH)D_3_ in cord blood among infants with different classification of NRDS. Data were shown with median with quarterback rang. One-way ANOVA followed Dunn's multiple comparisons test. **D** Spearman correlation analysis of 25(OH)D_3_ in cord blood and oxygenation index in preterm babies with neonatal respiratory distress syndrome

The level of 25(OH)D_3_ in cord blood was positively correlated with neonatal weight (Fig. 1A). However, it was not significantly associated with preterm birth time (Fig. 1B). To further explore the role of 25(OH)D_3_ in the NRDS of the preterm babies, we compared the difference in cord blood serum 25(OH)D_3_ level between the two groups. We found that the level of 25(OH)D_3_ in the cord blood serum of preterm infants with NRDS was significantly reduced compared with the control group (Fig. 2A). The sensitivity of 25(OH)D_3_ in cord blood serum was 76.83% and the specificity was 70.73%, respectively (57.69 as the cutoff value of 25(OH)D_3_). The area under curve (AUC) was 0.786 (Fig. 2B). ROC analysis revealed that 25(OH)D_3_ in the umbilical cord blood serum of preterm infants could predict NRDS. Then we divided the preterm infants with NRDS into subgroups according to the Montreux criteria of "Newborn Acute Respiratory Distress Syndrome" (2017 Edition). Our results revealed that the 25(OH)D_3_ level in cord blood serum gradually decreased with the increase of the severity of NRDS (Fig. 2C, *p*<<0.05). Finally, we demonstrated that there was a significant negative correlation between the oxygenation index (OI) and the 25(OH)D_3_ of the preterm infants (Fig. 2D, *p*<0.01). To further exclude the effect of asphyxia on the relationship between 25(OH)D_3_ and IO, we analyzed serum 25(OH)D_3_ levels in 61 neonates with NRDS but without asphyxia in Fig. S1. Spearman correlation analysis demonstrated that serum 25(OH)D_3_ levels in these neonates not affected by asphyxia were also statistically negatively correlated with OI.

### Monofactor analysis of NRDS risk factors

**Fig. 3:**
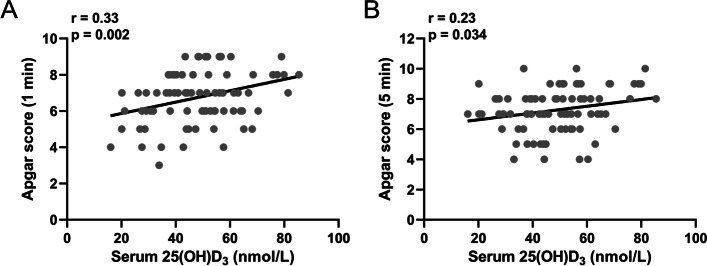
Spearman correlation analysis of 25(OH)D_3_ in cord blood with Apgar score (1 min) **A** and Apgar score (5 min) **B** in preterm babies with neonatal respiratory distress syndrome

**Fig. 4:**
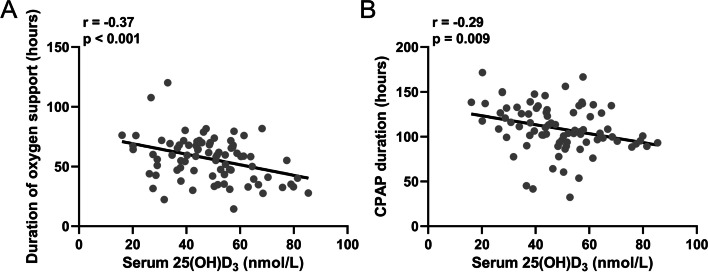
Spearman correlation analysis of 25(OH)D_3_ in cord blood with duration of oxygen support **A** and CPAP duration **B** in preterm babies with neonatal respiratory distress syndrome

We further analyzed the correlation between 25(OH)D_3_ level and Apgar score (1 min/5 min). There was a positive correlation between Apgar score and 25(OH)D_3_ level (Fig. 3A and B). Subsequently, we analyzed the correlation between the 25(OH)D_3_ in preterm infants and duration of oxygen support/ CPAP. Our results demonstrated that the level of 25(OH)D_3_ was significantly negatively correlated with oxygen support or CPAP duration (Fig. 4A and B), suggesting that 25(OH)D_3_ in the umbilical cord blood serum of preterm infants is related to the NRDS treatment status.

### Multivariate logistic regression analysis of NRDS risk factors

The regression model (the occurrence of NRDS was the dependent variable, and the variables in Table 3 were the independent variables) showed that cord blood serum 25(OH)D_3_<57.69 nmol/L (OR=4.784, 95% CI: 1.895-8.364, *p*=0.002), birth weight<1.86 kg (OR=2.216, 95% CI: 1.484-4.35, *p*=0.005), Apgar score (1 min) <7 (OR=6.216, 95% CI: 3.756-9.337, *p*<0.001), gestational age<31 weeks (OR=7.163, 95% CI: 4.075-12.547, *p*<0.001), and Apgar score (5 min) <8 (OR=1.967, 95% CI: 1.253-3.188, *p*=0.017) are independent risk factors for NRDS.

**Table 3:** Multivariate logistic analysis for neonatal respiratory distress syndrome

	OR	95% CI	*p* value
Serum levels of 25(OH)D_3_ in cord blood < 57.69 nmol/L	4.784	1.895 to 8.364	0.002
Gestational age < 31 weeks	7.163	4.075 to 12.547	< 0.001
Birth weight < 1.86 kg	2.216	1.484 to 4.35	0.005
Apgar score (1 min) < 7	6.216	3.756 to 9.337	< 0.001
Apgar score (1 min) < 8	1.967	1.253 to 3.188	0.017

*OR* Odds ratio, *CI* Confidence interval

## Discussion

NRDS is a common acute disease in pediatrics [11]. It has been reported that the onset of NRDS is closely related to lung hypoplasia, progressive alveolar collapse and high permeability lesions of pulmonary alveoli [12]. NRDS is a self-limiting disease [13]. However, there is a greater risk of concurrent infection during the onset of the NRDS, and NRDS can seriously threaten the lives of the infants. Therefore, exploring biochemical indicators that may be able to detect and judge the risk of NRDS is necessary.

Accumulating evidence has explained the association of vitamin D levels at birth and pulmonary disease morbidity in very preterm infants [14]. The use of vitamin D as adjuvant therapy in RDS cases resulted in significantly lower severity, complication rates, and length of hospital stay in preterm infants with RDS [15]. Barchita et al. reported that spontaneous and idiopathic preterm labor is associated with vitamin D deficiency. Preterm birth is an important factor leading to NRDS, and it was initially revealed that vitamin D deficiency caused by preterm birth may be related to NRDS [16, 17]. Decreased vitamin D is related to preterm birth, and vitamin D supplementation during pregnancy can reduce the rate of preterm birth [18, 19]. Aylor et al. reported that the levels of alveolar surfactant protein B and vascular endothelial growth factor increased through inhalation of vitamin D, suggesting that vitamin D might exhibit special functions in the management and treatment of NRDS [20]. Decreased vitamin D is a common and changeable risk factor for bronchopulmonary dysplasia and RDS [21]. A recent large prospective study which covered 402 newborn infants and their mothers suggested that cord blood vitamin D deficiency was associated with increased risk of preterm birth, NRDS and hospitalization during the first year of life [22]. Interestingly, some studies found no correlation between vitamin D and neonatal lung disease or lung co-development. For example, in a study that included 44 extremely preterm infants (gestational age <29 weeks), neither cord blood nor the 36 weeks' corrected age 25(OH)D_3_ levels were associated with development of bronchopulmonary dysplasia [23]. Furthermore, no significant association was found between vitamin D status and selected clinical outcomes when using a cut-off of 25 nmol/l (severe vitamin D deficiency) in preterm infants [24]. Considering a large number of animal and clinical studies that have revealed the importance of vitamin D for the development of the neonatal respiratory system, we believe that the differences in these results are because the cutoff values used in the above studies were not strong enough to distinguish differences in respiratory disease in preterm infants with vitamin D deficiency from other preterm infants.

There is still few clinical research on the effect of serum 25(OH)D_3_ concentration on the extra-skeletal effects of premature infants. It is still unclear how much serum 25(OH)D_3_ concentration can satisfy the needs of preterm infants. This study indicates that the 25(OH)D_3_ level in the serum of newborns with NRDS was statistically decreased than the control group, suggesting that the low 25(OH)D_3_ of newborns may be related to NRDS. The level of serum 25(OH)D_3_ of newborns could be used to predict NRDS taking 57.69 nmol/L (24ng/ml) as the cut-off value. In addition, we found that the serum 25(OH)D_3_ level in the cord blood serum of children was significantly positively correlated with Apgar score, and significantly negatively correlated with OI, duration of oxygen support and continuous positive airway pressure ventilation. The possible mechanism is that, lung surface active substances are produced by alveolar type II epithelial cells, which have vitamin D receptors and renal tubules 1α-hydroxylase protein expression. Therefore, alveolar type 2 epithelial cells may be the target cells for vitamin D bioregulation, which will have a certain impact. For the developing fetus, the lack of vitamin D leads to insufficient alveolar surface-active substances, thus affecting the development of lung structure and function. Therefore, 25(OH)D_3_ is related to NRDS in participants of our research.

In addition, in order to further analyze the relationship between neonatal 25(OH)D_3_ and NRDS, the risk factors of NRDS were analyzed. Our results suggested that cord blood serum 25(OH)D_3_ <57.69 nmol/L (24 ng/ml), gestational age<31 weeks, birth weight<1.86kg, Apgar score (1 min) <7 and Apgar score (5 min) <8 are independent risk factors for NRDS (*p*<0.05). Our data is consistent with the data of previous related studies. Decreased vitamin D in preterm infants is an independent risk factor for RDS [10]. In addition, Fettha et al. confirmed that higher serum 25(OH)D_3_ level in preterm infants could prevent RDS in preterm infants [25]. However, the difference is that in the latest prospective cohort study, when using 10 ng/ml as the cut-off value, vitamin D was not related to RDS, and the serum 25(OH)D_3_ level of infants did not affect the incidence of RDS [26]. This may be due to the use of 10 ng/mL as the cutoff value in the above-mentioned study was not sufficient to distinguish the difference between the two groups. At present, there is no consistent international standard for the optimal level of 25(OH)D_3_ in preterm infants [27]. Many scholars believe that 25(OH)D_3_ concentration <20 ng/ml indicates insufficient, and serum concentration<10 ng/ml indicates severe deficiency [26, 28, 29]. The cut-off value in this study is 57.69 nmol/L (24 ng/ml), which suggests that we may need a higher serum 25(OH)D_3_ level to prevent NRDS. However, patients who take vitamin D excessively for a long time may suffer from vitamin D poisoning, when the content of 25(OH)D_3_ in their blood exceeds 100ng/ml. Although vitamin D toxicity is rare in clinical practice, we should actively monitor the 25(OH)D_3_ level in patients' blood during treatment.

In fact, there are still many deficiencies in our research. First, due to the limitations of hospital size and department beds, we only recruited 82 qualified infants with NRDS. Insufficient number of infants may affect the accuracy of statistical results. We plan to conduct future multicenter joint studies to expand the number of patients with NRDS. Second, we mainly analyzed the relationship between vitamin D in umbilical cord blood and the incidence and treatment of postnatal NRDS. However, there is no reliable study to explain why vitamin D deficiency increases the NRDS incidence rate. The key role of vitamin D in the formation of fetal alveolar cells and the establishment of lung tissue structure remains unclear. We would like to further reveal the important role of vitamin D in fetal lung development in future mechanism research. In addition, the risk factors of NRDS revealed in this study are very limited. We only analyzed the relationship and correlation between 25(OH)D_3_ level, pregnancy time, birth weight, Apgar score (1 min) and NRDS in this study. However, previous studies have shown that the risk of NRDS may be due to maternal diabetes, fetal gender, delivery time, and difficult labor experience. Due to the limitation of manpower and information integrity, we did not analyze the relationship between these factors and NRDS incidence rate in this study. We intend to fully cover these factors in the future. Moreover, our study also lacked data on the association of cord blood 25(OH)D_3_ levels with subsequent incidence of neonatal bronchopulmonary dysplasia, and the association of cord blood 25(OH)D_3_ levels with maternal vitamin D status. We will continue to refine these studies in future research.

## Conclusion

In conclusion, we demonstrated that serum 25(OH)D_3_ level in the premature newborns were negatively correlated with the occurrence of NRDS. Moreover, serum 25(OH)D_3_ level in the newborn is also an independent risk factor for NRDS. Our study reveals that the monitoring of serum 25(OH)D_3_ levels in clinically preterm neonates may play a predictive role in the occurrence of NRDS.

## Supplementary Information

**Additional file1: Supplementary Table S1.** Comparison of hospitalization of the preterm babies with (NRDS) and without (Control) neonatal respiratory distress syndrome. **Supplementary Figure S1.** In 61 infants with NRDS but not affected by asphyxia, spearman correlation analysis of 25(OH)D3 in cord blood with oxygenation index (OI).

## Data Availability

The datasets used and/or analysed during the current study are available from the corresponding author on reasonable request.

## Abbreviations

NRDS: Neonatal respiratory distress syndrome
ROC: Receiver operating characteristics curve
CPAP: Continuous positive airway pressure ventilation
OI: Oxygenation index
AUC: Area under curve
