# Supplementary material for: The association of serum vitamin D level and neonatal respiratory distress syndrome
Source: Ital J Pediatr. 2023 Jan 30;49:16. doi: 10.1186/s13052-023-01415-w (PMC9885703; doi:10.1186/s13052-023-01415-w)
Supplement: Supplementary file 1 — Additional file1: Supplementary Table S1. Comparison of hospitalization of the preterm babies with (NRDS) and without (Control) neonatal respiratory distress syndrome. Supplementary Figure S1. In 61 infants with NRDS but not affected by asphyxia, spearman correlation analysis of 25(OH)D3 in cord blood with oxygenation index (OI). [file 13052_2023_1415_MOESM1_ESM.docx]

**Supplementary materials**

Supplementary Table S1. Comparison of hospitalization of the preterm babies with (NRDS) and without (Control) neonatal respiratory distress syndrome.

| Characteristics | Study group | | p value |
| --- | --- | --- | --- |
|  | Control  (n=82) | NRDS  (n=82) |  |
| CPAP duration (hours) | 27.4±8.4 | 108.5±27.9 | < 0.001 |
| Duration of oxygen support (hours) | 15.1±5.9 | 56.5±18.3 | < 0.001 |
| Length of hospital stay (days) | 6.2±2.8 | 16.6±3.6 | < 0.001 |
| Venous nutrition duration (days) | 5.7±3.1 | 12.8±2.9 | < 0.001 |

Values were expressed as mean ± SD. p values for each group were derived from Mann–Whitney test.

CPAP: continuous positive airway pressure ventilation


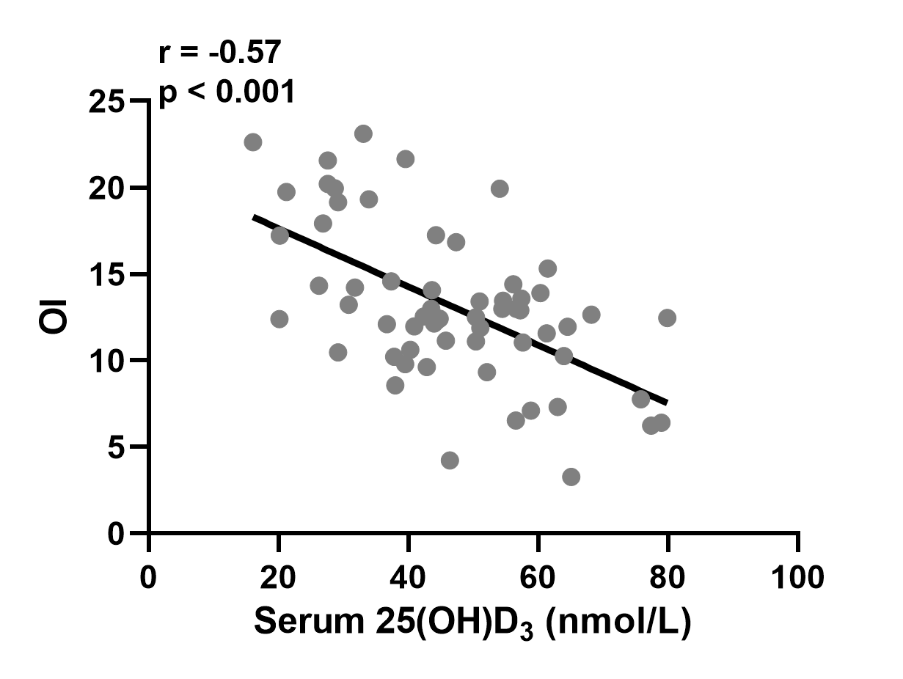

**Supplementary Figure S1**. In 61 infants with NRDS but not affected by asphyxia, spearman correlation analysis of 25(OH)D3 in cord blood with oxygenation index (OI).
